# Cytochrome *P*450 CYP1B1 activity in renal cell carcinoma

**DOI:** 10.1038/sj.bjc.6602053

**Published:** 2004-07-27

**Authors:** M C E McFadyen, W T Melvin, G I Murray

**Affiliations:** 1 Department of Pathology, University of Aberdeen, Foresterhill, Aberdeen AB25 2ZD, UK; 2 Department of Molecular and Cellular Biology, University of Aberdeen, Foresterhill, Aberdeen AB25 2ZD, UK

**Keywords:** CYP1B1, P450 reductase, renal cell carcinoma, tumour, therapeutic intervention

## Abstract

Renal cell carcinoma (RCC) is the most common malignancy of the kidney and has a poor prognosis due to its late presentation and resistance to current anticancer drugs. One mechanism of drug resistance, which is potentially amenable to therapeutic intervention, is based on studies in our laboratory. CYP1B1 is a cytochrome *P*450 enzyme overexpressed in a variety of malignant tumours. Our studies are now elucidating a functional role for CYP1B1 in drug resistance. Cytochrome *P*450 reductase (P450R) is required for optimal metabolic activity of CYP1B1. Both CYP1B1 and P450R can catalyse the biotransformation of anticancer drugs at the site of the tumour. In this investigation, we determined the expression of CYP1B1 and P450R in samples of normal kidney and RCC (11 paired normal and tumour and a further 15 tumour samples). The O-deethylation of ethoxyresorufin to resorufin was used to measure CYP1B1 activity in RCC. Cytochrome *P*450 reductase activity was determined by following the reduction of cytochrome *c* at 550 nm. The key finding of this study was the presence of active CYP1B1 in 70% of RCC. Coincubation with the CYP1B1 inhibitor alpha-naphthoflavone (10 nM) inhibited this activity. No corresponding CYP1B1 activity was detected in any of the normal tissue examined (*n*=11). Measurable levels of active P450R were determined in all normal (*n*=11) and tumour samples (*n*=26). The presence of detectable CYP1B1, which is capable of metabolising anticancer drugs in tumour cells, highlights a novel target for therapeutic intervention.

Renal cell carcinoma (RCC) is the most common malignancy of the kidney accounting for 85% of all tumours of the kidney (Gnarra *et al*, 1995). Currently, there are approximately 550 new cases of RCC diagnosed in Scotland annually with 320 deaths from this disease in the same time period. Unfortunately, RCC is curable only in patients presenting with resectable early-stage disease.

The 5-year survival of patients with kidney tumours is only 43% (ISD, 2004) and this poor prognosis is associated with late presentation and poor response to standard anticancer drugs. Moreover, the median survival of patients presenting with locally advanced or metastatic disease is less than 1 year (Lineham *et al*, 1997). Although RCC is a highly vascular tumour and extracellular drug delivery is probably high (Yagoda *et al*, 1995), chemotherapy is largely ineffective, and response rates to cytotoxic therapy are only 5–15% (Chapman and Goldstein, 1995). Indeed, the response of RCC to the alkylating agents (nitrosoureas, melphalan, cyclophosphamide and ifosphamide) is very low (Gattinoni *et al*, 2003). Docetaxel, vinblastine and the 5′-fluoropyrimidines have all been highlighted as effective single agents for metastatic disease, but the response rate is still only 20% (Gattinoni *et al*, 2003). Resistance of RCC to anticancer drugs is considered to be due in part to the activity of the superfamily of drug efflux pumps, which are responsible for ‘classical’ multidrug resistance; p-glycoprotein (also known as MDR1 (multidrug resistance) in human beings) was the first of this superfamily to be identified. Several treatments including agents that alter MDR (acrivastine, nifedipine, cyclosporin A and other derivatives such as PSC833, quinidine and verapamil) and immunotherapy (using interleukin 2 and interferon *α*) have been proposed as prospective new therapies for RCC; however, the results have been disappointing (Gattinoni *et al*, 2003). Indeed, cancers of the kidney are considered to be among the most unresponsive to treatment and intrinsically drug resistant of all human cancers (Yagoda *et al*, 1995), and RCC remains fatal in nearly 80% of patients.

One mechanism of drug resistance, which is potentially amenable to therapeutic intervention, is based on studies in our laboratory. CYP1B1 is a cytochrome *P*450 xenobiotic metabolising enzyme, which is central to the oxidative metabolism of a wide variety of endogenous and exogenous compounds. In contrast, to other cytochrome *P*450 enzymes, CYP1B1 is not expressed in human liver (Murray *et al*, 1997; Edwards *et al*, 1998), but demonstrates enhanced expression of immunoreactive protein in a wide range of histologically diverse tumours including kidney, ovarian, breast and colon tumours (Murray *et al*, 1997; McFadyen *et al*, 1999, 2001a; Gibson *et al*, 2003). Our previous *in vitro* studies have shown that CYP1B1 is specifically localised to tumour cells, with no concomitant expression in normal tissue (Murray *et al*, 1997; McFadyen *et al*, 1999, 2001a).

Several P450's have an established role in the metabolic biotransformation of a wide variety of substrates including a number of procarcinogens and several anticancer drugs (Gut *et al*, 2000; Kajita *et al*, 2000; Fujita and Kamataki 2001; Stiborova *et al*, 2001; Yamazaki *et al*, 2001; Lewis *et al*, 2003; Schwarz and Roots, 2003). Metabolic activation of procarcinogens and promutagens can result in the accumulation of damaging DNA adducts; Shimada and co-workers have shown CYP1B1 bioactivation of a diverse range of these compounds including carcinogenic polycyclic and nitro aromatic hydrocarbons and arylamines (Shimada *et al*, 1996, 1997).

Our *in vitro* studies have identified several anticancer drugs (docetaxel, paclitaxel, mitoxantrone and flutamide) as substrates for CYP1B1 (Rochat *et al*, 2001). Furthermore, we have shown that the presence of CYP1B1 reduces the efficacy of docetaxel. Indeed, a recent study has confirmed that CYP1B1 interacts with docetaxel and reduces its cytotoxicity (Bournique and Lemarie, 2002).

We have previously highlighted the use of CYP1 inhibitors in modulating the cytotoxic profile of a range of structurally diverse anticancer drugs with CYP1B1 (McFadyen *et al*, 2001b; Rochat *et al*, 2001; McFadyen *et al*, 2004). Chang and co-workers recently demonstrated that the stilbene *trans*-resveratrol inhibited the catalytic activity of CYP1B1 (Chang *et al*, 2000).

An inherent problem in determining CYP1B1 activity in tumour tissue is that there are no truly specific assays to distinguish between CYP1A1, CYP1A2 and CYP1B1 activity, which show close homology with one another and overlapping substrate specificity. However, the CYP1 inhibitor alpha-naphthoflavone has been shown to be a suitable inhibitor to distinguish between CYP1A1 and CYP1B1 activity, and at low concentration, that is, 10 nM alpha-naphthoflavone inhibits CYP1B1 activity by 80% and CYP1A1 activity by only 20% (Shimada *et al*, 1998). CYP1A2 is constitutively expressed in the liver and is not found in extrahepatic tissues. In this study, we used alpha-naphthoflavone in conjunction with the O-deethylation of ethoxyresorufin, a model substrate for the CYP1 family of enzymes to determine CYP1B1 activity.

The ability to inhibit CYP1B1 activation may have important clinical implications both in reducing drug resistance by increasing the efficacy of anticancer drugs and preventing procarcinogen activation.

Cytochrome *P*450 enzymes require the presence of the microsomal flavoprotein cytochrome *P*450 reductase (P450R) for optimal metabolic activity (Lu and Coon 1968; Lu *et al*, 1969). The main function of this enzyme is the transfer of electrons from NADPH via FAD and FMN cofactors to cytochrome *P*450 enzymes (Vermilion *et al*, 1981). Cytochrome *P*450 reductase in conjunction with the cytochrome *P*450 enzymes is central to the oxidative metabolism of a wide range of anticancer drugs.

Therefore, to have a fuller understanding of the importance of CYP1B1 as a therapeutic target in tumours, it is important to determine the level of active CYP1B1 and P450R.

## MATERIALS AND METHODS

### Tissue

Samples of 11 paired normal kidney and kidney tumour and 15 additional kidney tumours submitted to the Department of Pathology, University of Aberdeen for diagnosis over a 4-year period were used in this study. The samples collected in this study were consecutive cases of RCC and no special case selection was made. None of the patients had received chemotherapy prior to surgery. All the tumours were primary renal cell cancers and histological classification of the tumours performed according to the current criteria (Kovacs *et al*, 1997) showed 23 clear-cell carcinomas, two chromophobe carcinomas and one papillary carcinoma. The clinicopathological data for each patient are summarised in Table 1

**Table 1:** Clinicopathological information of patients with RCC

Age (median and range)	63 years (35–96 years)
	
*TNM stage of renal cell carcinoma*	
1	6/26 (23%)
2	10/26 (38.5%)
3	10/26 (38.5%)
	
*Histological subtype*	
Clear-cell carcinoma	23/26 (88%)
Chromophobe	2/26 (8%)
Papillary	1/26 (4%)

RCC=renal cell carcinoma.

. Samples of tumour and normal kidney were dissected from the individual nephrectomy specimens. Connective, haemorrhagic and necrotic tissue and fat were removed from the individual specimens and samples of tumour were taken from areas of viable tumour (confirmed by histological examination of haematoxylin- and eosin-stained sections). Corresponding samples of normal kidney were taken at least 5 cm from the tumour and confirmed by histological examination to be composed of normal kidney with no contamination by tumour cells. The dissected samples were then stored at −70°C prior to use, we have previously shown that this method of sampling has no adverse effect on either protein (Murray *et al*, 1999) or RNA (Cheung *et al*, 1999) analysis. Ethical approval for this project was obtained from Grampian research ethics committee.

### Preparation of microsomes

Frozen samples of normal kidney and RCCs were thawed on ice in 0.01 M. Tris-HCl pH 7.4 containing 1.15% potassium chloride. Once thawed, the normal and tumour tissue were homogenised in 0.01 M Tris-HCl containing 0.25 M sucrose and 15% glycerol using a Polytron PT3000 Homogeniser (Kinematica AG, Switzerland). Homogenates were centrifuged at 15 000 **g** for 20 min at 4°C using a Centrikon T-124 centrifuge (Kontron Instruments, Cumbernauld, UK). The resultant supernatants were then centrifuged at 180 000 **g** (44 000 r.p.m.) for 1 h at 4°C using a Centrikon T-1160 centrifuge (Kontron Instruments). The pellet obtained after centrifugation was resuspended in 0.1 M Tris-HCl, containing 15% glycerol and 1 mM EDTA, and centrifuged again at 180 000 **g** (44 000 r.p.m.) for 1 h at 4°C. A final pellet was obtained and resuspended in 0.1 M Tris-HCl containing 15% glycerol and 1 mM EDTA. The resultant microsomes were stored at −75°C prior to use. Protein concentrations for each sample of microsomes were determined using the Bradford method (Bradford, 1976).

### Validation of O-deethylation of ethoxyresorufin assay for determining CYP1B1 activity

The dealkylation of the alkoxyphenazone compound ethoxyresorufin, a model substrate for the CYP1 family of P450 enzymes, was used as a probe for CYP1B1 activity in this study.

To validate this assay as a measure of CYP1B1 activity, the production of resorufin from the O-deethylation of ethoxyresorufin by both CYP1B1 and CYP1A1 was measured. Microsomes prepared from human lymphoblastoid cells, that contained either expressed human CYP1A1 or CYP1B1, were obtained from Gentest (Gentest Corporation, Cambridge Bioscience Ltd, Cambridge, UK) and used in these experiments. Serial concentrations of the CYP1 inhibitor alpha-naphthoflavone were included at the following concentrations 0, 0.1, 0.5, 5, 10, 50 and 100 nM. Briefly, each 2 ml incubation contained, either CYP1A1 (1 pmol) or CYP1B1 (5 pmol) microsomes, 100 mM Tris-HCl pH 7.4 incubated in 2 ml reaction volume of Tris buffer containing NADPH-regenerating system (1.3 mM NADP^+^, 3.3 mM glucose-6-phosphate, 0.4 U ml^−1^ glucose-6-phosphate dehydrogenase, 3.3 mM magnesium chloride) for 10 min at 37°C prior to the addition of ethoxyresorufin (5 *μ*M final concentration) with or without the addition of alpha-naphthoflavone. The production of resorufin was measured by scanning spectrophotometer at the following wavelengths: excitation 530 nm and emission 585 nm. Readings were taken immediately following the addition of ethoxyresorufin continuously over a 40 min time period. The activity was calculated from the linear portion of the graph against a known resorufin standard (100 pM).

### Efficiency of CYP1B1 O-deethylation of ethoxyresorufin

To evaluate the optimal conditions and efficiency of the reaction, we determined the *K*_m_ (substrate concentration at half the maximum velocity) of the reaction. This was performed using serial substrate concentrations, that is, ethoxyresorufin was added at the following final concentrations 0.1, 0.25, 0.5, 1, 2.5, 3.75, 5, 7.5, 10 and 12.5 *μ*M.

### CYP1B1 activity: O-deethylation of ethoxyresorufin

The production of resorufin from the O-deethylation of ethoxyresorufin was determined by a modified continuous spectrofluorimetric assay based on the method of Burke and Mayer (1974). Briefly, each 2 ml incubation contained 100 mM Tris-HCl pH 7.4, NADPH-regenerating system and the microsomal fraction of tumour samples (1 mg protein); this mixture was incubated with or without the addition of 10 nM alpha-naphthoflavone for 10 min at 37°C prior to the addition of ethoxyresorufin (1 *μ*M final concentration). The production of resorufin was measured by scanning spectrophotometer at the following wavelengths: excitation 530 nm and emission 585 nm. Readings were taken over a 40-min period immediately following the addition of EROD. The activity was calculated from the linear portion of graph against a known resorufin standard (100 pM). To take account of day-to-day variation of the assay system, CYP1A1 and CYP1B1 microsomes were included in all assays as positive controls.

### P450R activity

As the reduction of P450R is relatively difficult to measure directly, a simplified determination of enzyme activity is widely used utilising exogenous cytochrome *c* (oxidised, ferric form) as an artificial electron acceptor. Accordingly, the reduction of cytochrome *c* by NADPH–cytochrome *c* (P450) reductase mirrors the reduction P450R. The principles of this are that the reduced (ferrous) form has a characteristic absorption band at 550 nm, which is absent in the oxidised (ferric) form of cytochrome *c*. Therefore, the enzyme activity can be conveniently assayed by measuring the increase in absorbance at 550 nm.

Briefly, each 2 ml incubation contained 1.3 mM NADP^+^, 3.3 mM glucose-6-phosphate, 0.4 U ml^−1^ glucose-6-phosphate dehydrogenase, 3.3 mM magnesium chloride and 1 mg ml^−1^ cytochrome *c* in 0.1 M Tris-HCl (pH7.4); this mixture was incubated at 37°C prior to the addition of 100 *μ*g of microsomal protein. As the tissue samples had a high turbidity, a split-beam spectrophotometer comparing a sample cuvette with a reference cuvette, which contained no regenerating system, was used to control for sample turbidity.

Readings were taken continuously following the addition of the microsomal protein over a 40 min time period. The change in absorbance was calculated automatically as a function of time for the linear period of the reaction. An extinction coefficient for reduced (ferrous) cytochrome *c* at 550 nm of 19.6 mM^−1^ cm^−1^ was used to calculate the reductase activity.

### Statistical analysis

Statistical analysis was performed using both SigmaStat 2.03 for Windows 2000™ and SPSS version 11.5 for Windows 2000™.

### Data analysis: coefficient of variation (CV)

All assays were performed in triplicate. The mean and standard deviation were calculated for each of the samples, which were then further analysed to determine the CV. Based on our experience with the assays, a CV of 10% was accepted as the maximum variation allowed under the conditions used, any sample out with this value was discarded and repeated.

## RESULTS

### Validation of O-deethylation of ethoxyresorufin assay for determining CYP1B1 activity

**Figure 1:**
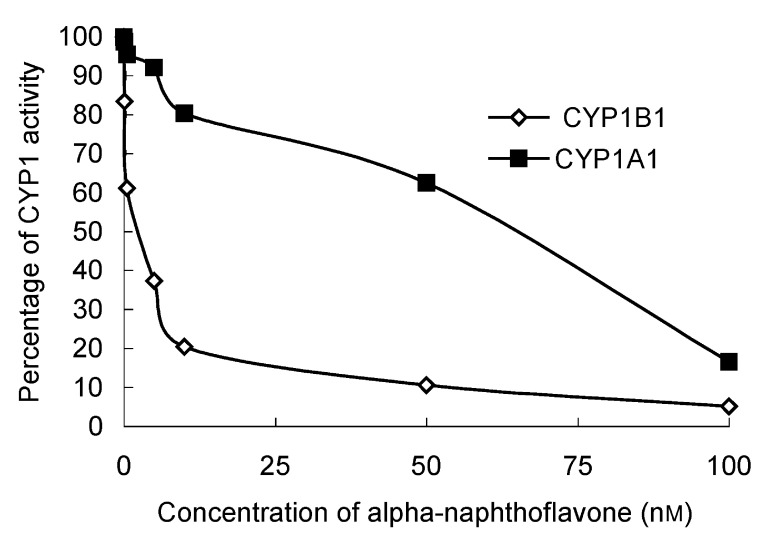
Graphs illustrating the percentage of CYP1 activity demonstrating the difference in CYP1B1 and CYP1A1 activity (production of resorufin from the O-deethylation of ethoxyresorufin) following the addition of alphanaphthoflavone (0, 0.1, 0.5, 5, 10, 50 and 100 nM final concentration). All assays were performed in triplicate with CYP1A1 (1 pmol) and CYP1B1 (5 pmol) containing microsomes prepared from either CYP1A1- or CYP1B1-expressing human lymphoblastoid cells, respectively.

Determination of CYP1B1 activity was accomplished by measuring resorufin production from the O-deethylation of ethoxyresorufin. However, as ethoxyresorufin is also a substrate for CYP1A1 we modified our assay system to distinguish between CYP1A1 and CYP1B1 activities in tumour tissue, by performing the reaction with low concentrations (10 nM) of alpha-naphthoflavone, which markedly inhibited CYP1B1 activity by 80% and inhibited CYP1A1 to a much lesser (<20%) extent (Figure 1

).

### Efficiency of CYP1B1 O-deethylation of ethoxyresorufin

**Figure 2:**
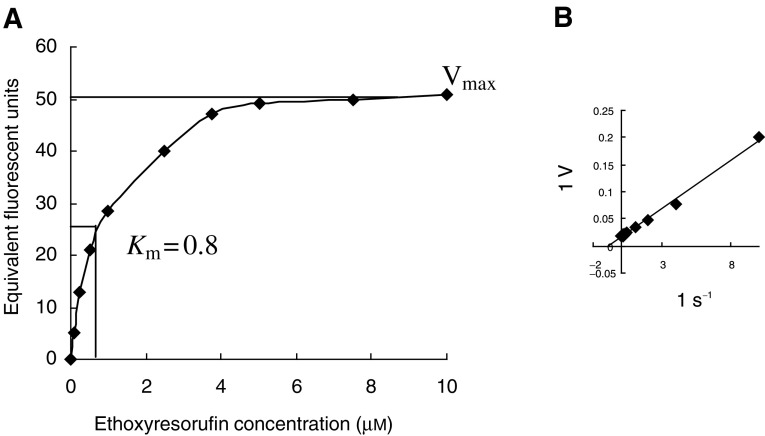
Efficiency of CYP1B1 activity demonstrated by (**A**) Michaelis–Menten and (**B**) Linneweaver–Burke plots illustrating the *K*_m_ and *V*_max_ for CYP1B1 O-deethylation of ethoxyresorufin.

The ethoxyresorufin activity of the lymphoblastoid expressed CYP1B1 was determined at various concentrations of substrate. The *K*_m_ of the reaction was determined as 0.8 *μ*M (Figure 2

).

### CYP1B1 activity: O-deethylation of ethoxyresorufin

**Figure 3:**
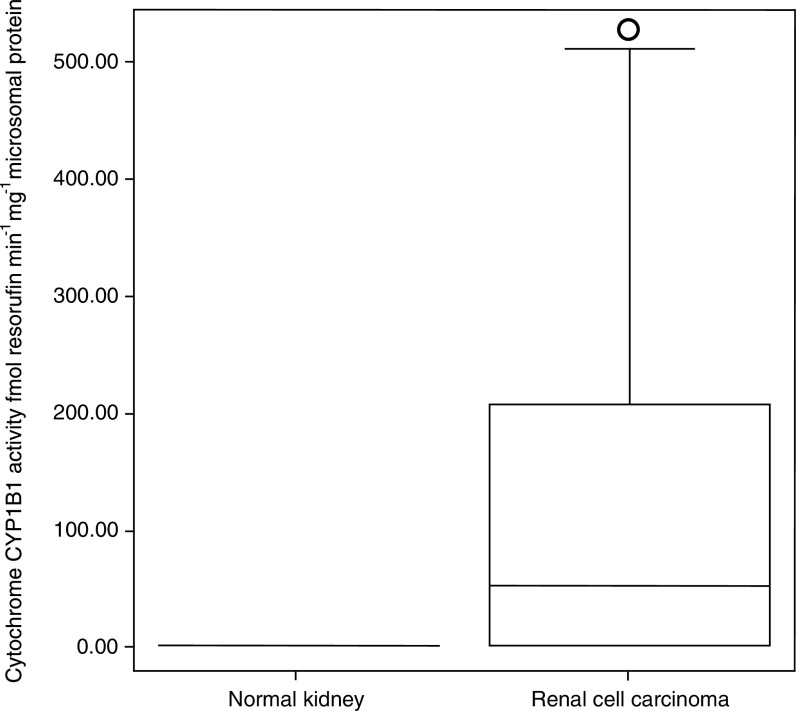
Distribution of CYP1B1 activity in normal kidney and RCC. CYP1B1 activity according to the change in resorufin production Δ*R*=*R*_*ni*_−*R*_*i*_ (Δ*R* is the change in CYP1B1 activity, *R*_*ni*_ is the resorufin production in the absence of the inhibitor alpha-naphthoflavone and *R*_*i*_ is the resorufin production in the presence of 10 nM alpha-naphthoflavone. Boxes delimit the first and third quartiles with the median inside. Circle (○) represents outlier, defined as individual values 1.5–3 times greater than the interquartile range. No box is present for the normal kidney due to the absence of CYP1B1 activity.

Determination of CYP1B1 activity was accomplished by measuring resorufin production from the O-deethylation of ethoxyresorufin in the presence or absence of 10 nM alpha-naphthoflavone. EROD activity was identified in 82% of clear-cell renal carcinomas (*n*=19/23) (65–992 fmol min mg^−1^ of microsomal protein) with similar levels of CYP1B1 activity also demonstrated in the chromophobe (*n*=2) and papillary renal carcinomas (*n*=1). Coincubation with the CYP1B1 inhibitor alpha-naphthoflavone at 10 nM partially inhibited this activity by greater than 20% in 16 out of the 19 (70%) RCCs demonstrating the O-deethylation of ethoxyresorufin (Figure 3

). No production of resorufin (CYP1B1 activity) was detectable in any of the normal tissue samples (Figure 3). The limit of sensitivity of this assay was 20 fmol min mg^−1^ of microsomal protein.

### Cytochrome *P*450 reductase

**Figure 4:**
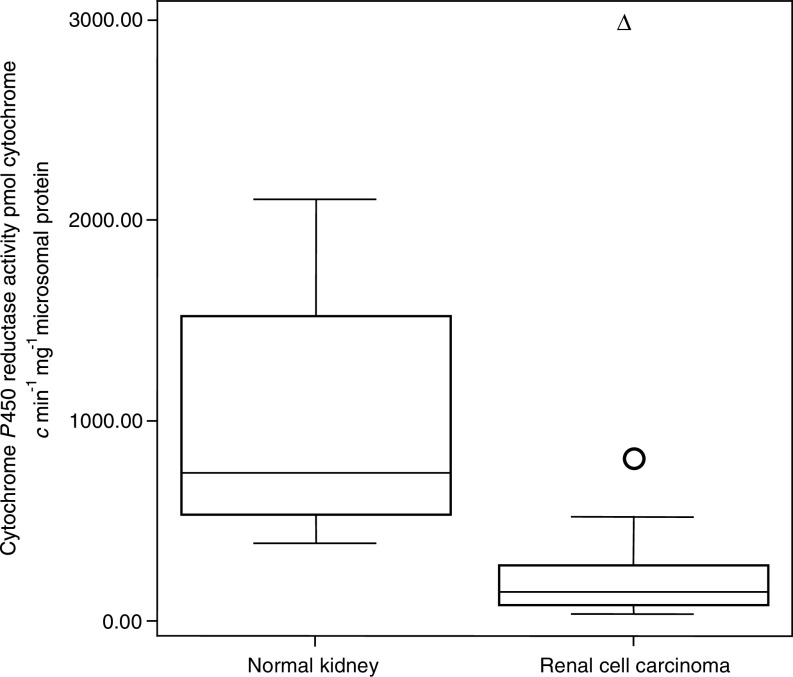
Distribution of P450R activity in normal kidney and RCC, according to cytochrome *c* production. Boxes delimit the first and third quartiles, with the median inside and bars representing the range of values that fall within 1.5-fold the interquartile range. Circle (○) represents outlier, defined as individual values 1.5–3 times greater than the interquartile range. Triangle (Δ) represents extreme, defined as individual values greater than three times the interquartile range.

All the normal tissue and tumour samples demonstrated P450R activity with considerable variability in expression between the two groups. A higher level of P450R was demonstrated in the normal kidney (mean=1024 and range=525–2097 P450R activity pmol min mg^−1^ microsomal protein) compared to the kidney tumours (mean=308 and range=34–2969 P450R activity pmol min mg^−1^ microsomal protein) Figure 4

. This difference in P450R activity between the normal kidney and RCC was shown to be significant in the 11 paired samples by a paired *t*-test (*t*=4.614, degrees of freedom (df)=10, *P*=0.001). No relationship was found between P450R activity histological diagnosis and stage of the tumours (*χ*^2^=2.889, with 4 df, *P*=0.577). The limit of sensitivity of this assay was 10 pmol of P450R activity min mg^−1^ microsomal protein.

## DISCUSSION

CYP1B1 has been identified in a wide range of histologically distinct human neoplasms. Our *in vitro* studies have shown that this P450 is overexpressed in tumours and is specifically expressed in tumour cells (Murray *et al*, 1997; McFadyen *et al*, 1999; Murray, 2000). We have recently shown that CYP1B1 demonstrates a similarly high level of expression in metastatic disease (McFadyen *et al*, 2001a). Moreover, the presence of CYP1B1 protein is undetectable in normal liver (Murray *et al*, 1997; Edwards *et al*, 1998; Chang *et al*, 2003). In this study, by the use of the CYP1 inhibitor alpha-naphthoflavone at a low concentration (10 nM), which distinguishes between CYP1A1 and CYP1B1, we showed measurable CYP1B1 activity in RCC. We found CYP1B1 activity in primary RCC in 16 out of 19 samples (70%) with no detectable CYP1B1 observed in the normal kidney. However, we were unable to inhibit all the P450 activity observed in the RCCs at the concentration of alpha-naphthoflavone used in this study. Therefore, the presence of other metabolically active P450 enzymes such as CYP1A1 and CYP3A, which also catalyse the O-deethylation of ethoxyresorufin must be considered. This investigation has provided evidence for CYP1B1 activity in RCCs and the presence of other metabolically active P450 enzymes in RCCs.

Cytochrome P450 reductase is an essential component of the cytochrome *P*450 monoxygenase system, which catalyses the transfer of electrons from NADPH to P450 enzymes (Djordjevic *et al*, 1995). P450R is also involved in the bioreductive metabolism of a number of anticancer drugs including tirapazamine (Patterson *et al*, 1995). In this study, P450R was expressed in both normal and tumour tissue with some variation in expression observed in both groups. López de Cerain *et al* (1999) observed similar levels of P450R expression in normal lung and lung tumours. In this study, a higher level of P450R was observed in the normal tissue compared to the RCC. Henderson *et al* (2003) have recently reported an almost five-fold increase in P450 levels in P450R knockout mice, although the mechanism of action is unknown. Little is known about the regulatory mechanisms governing P450 enzymes in tumour tissue, and much remains to be elucidated about both the function and the mechanisms regulating their expression.

The presence of metabolically active CYP1B1, which is overexpressed in tumours and which is capable of metabolising anticancer drugs within the tumour cells offers tremendous opportunities for the development of novel prodrugs activated by CYP1B1 only in the tumour cells (Murray *et al*, 2001; Patterson and Murray, 2002). Indeed, several classes of prodrug, designed to be activated selectively by CYP1B1 at the site of the tumour, are currently in preclinical evaluation. One of the most promising of these compounds resveratrol was recently shown to be metabolised by CYP1B1 to the anticancer agent piceatannol (Potter *et al*, 2002).

In addition, the ability to inhibit CYP1B1 activity highlights a target for the development of an inhibitor, which would increase the efficacy of currently available cytotoxic agents at the site of the tumour.
